# Adaptation and evaluation of the bottle assay for monitoring insecticide resistance in disease vector mosquitoes in the Peruvian Amazon

**DOI:** 10.1186/1475-2875-8-208

**Published:** 2009-09-03

**Authors:** Elvira Zamora Perea, Rosario Balta León, Miriam Palomino Salcedo, William G Brogdon, Gregor J Devine

**Affiliations:** 1 Laboratorio de Salud Pública, Iquitos, Perú; 2 Instituto Nacional de Salud, Lima, Perú; 3 Centers for Disease Control, Atlanta, GA 30333, USA; 4 Rothamsted Research, Harpenden, AL5 2JQ, UK

## Abstract

**Background:**

The purpose of this study was to establish whether the "bottle assay", a tool for monitoring insecticide resistance in mosquitoes, can complement and augment the capabilities of the established WHO assay, particularly in resource-poor, logistically challenging environments.

**Methods:**

Laboratory reared *Aedes aegypti *and field collected *Anopheles darlingi *and *Anopheles albimanus *were used to assess the suitability of locally sourced solvents and formulated insecticides for use with the bottle assay. Using these adapted protocols, the ability of the bottle assay and the WHO assay to discriminate between deltamethrin-resistant *Anopheles albimanus *populations was compared. The diagnostic dose of deltamethrin that would identify resistance in currently susceptible populations of *An. darlingi *and *Ae. aegypti *was defined. The robustness of the bottle assay during a surveillance exercise in the Amazon was assessed.

**Results:**

The bottle assay (using technical or formulated material) and the WHO assay were equally able to differentiate deltamethrin-resistant and susceptible *An. albimanus *populations. A diagnostic dose of 10 μg a.i./bottle was identified as the most sensitive discriminating dose for characterizing resistance in *An. darlingi *and *Ae. aegypti*. Treated bottles, prepared using locally sourced solvents and insecticide formulations, can be stored for > 14 days and used three times. Bottles can be stored and transported under local conditions and field-assays can be completed in a single evening.

**Conclusion:**

The flexible and portable nature of the bottle assay and the ready availability of its components make it a potentially robust and useful tool for monitoring insecticide resistance and efficacy in remote areas that require minimal cost tools.

## Background

The countries of the Amazon basin (Bolivia, Brazil, Colombia, Ecuador, Guyana, Peru, Surinam and Venezuela) carry the greatest burden of malaria and arbovirus transmission in the Americas. In 2006, there were ca. 800,000 cases of malaria and ca. 400,000 cases of dengue in the region [1,2]. Mortality is low but the rising number of deaths from dengue haemorrhagic fever, and the chronic health and economic problems caused by relapsing malarias are of major concern. The primary vectors of these diseases in the Amazon basin are, respectively, *Aedes aegypti *and *Anopheles darlingi. Anopheles albimanus *is the main vector of malaria on Peru's Pacific coast [3].

Vector control is a key part of the management of arthropod-borne disease and insecticides remain the mainstay of most vector control programmes. When no vaccine, prophylaxis or treatment exist, it is the only option available (e.g. for preventing dengue outbreaks). The insecticidal tools employed include adulticides for indoor residual spraying (IRS), fumigation and space sprays, larvicides for the treatment of breeding sites, and the impregnation of bed nets and other materials for personal protection. Insecticide-based vector control is effective if optimally executed [4-7], but common barriers to implementation include limited local resources, poor operational capacity, the use of chemical classes that are resisted, and the application of adulterated insecticides. Simple bioassays, if carefully applied and calibrated, are the cheapest way to help identify many of these factors. Ideally they will characterize insect responses to different insecticide formulations, identify the presence of resistant populations and, by default, implicate application or equipment error as a cause of field control failures.

The world's standard assay for assessing resistance in mosquitoes is the WHO assay, introduced in 1958 [8]. After decades of use, its standardized protocol allows the comparison of results between laboratories, regions and sampling dates. However, its use in intensive monitoring exercises, particularly in remote regions where there is little money for implementation, is complicated by the requirement to purchase equipment and insecticides from a single source, and by the limited set of insecticides and concentrations available. The WHO assay might be complemented by a cheap, portable and flexible assay that could be easily deployed by local health posts. The "bottle assay", introduced by the Centers for Disease Control (CDC) in 1998 [9] may be suited for that purpose. Despite the fact that both the bottle and WHO assays are referred to in the literature [10-12], there are no comparative studies that might help local health authorities decide whether the bottle assay is a truly robust addition to the tools already available. This paper aims to provide that information.

## Methods

All laboratory work was conducted at the Laboratorio de Salud Pública, Iquitos, Loreto, Perú (3°44'S 73°15'W). The surveillance exercise took place in the department of Loreto, in the villages of Ullpayacu (4°38'S 76°35'W), Intuto (3°31'S 74°44'W), Libertad (3°29'S 73°14'W), and Zungarococha (3°49' S, 73°21' W). The comparison of assays was undertaken in the department of Piura, using mosquitoes from Maran (4°45'S 80°31'W) and Paimas (4°37'S 79°55'W).

### Evaluation of locally-sourced solvents and formulations

The dose-mortality responses of a colony of *Ae. aegypti *described previously [13] were compared using technical deltamethrin (> 98% purity, Sigma Aldrich) and a 2.5% a.i. wettable powder deltamethrin formulation (K-Othrine 25 WP, Bayer). A number of deltamethrin formulations are registered in Peru for vector control. Bottles were prepared as described by Brogdon and McAllister [9], except that the recommended solvent, technical grade acetone (> 99%, Sigma Aldrich), was compared with a cheap locally-available alternative (95% ethanol from local pharmacies). Illustrations of general techniques for the preparation of the bottles are available on the CDC website [14]. For all combinations of formulations and solvents, mortality was observed over four different, sub-LC_100 _insecticide doses (0.016, 0.08, 0.4, 2 μg a.i/bottle) and a 2 h period (15, 30, 45, 120 min). Each assay consisted of three bottles for each dose and control. Fifteen three- to four-day old females, starved of a blood meal for at least 24 h were exposed in each bottle. Each assay was repeated at least eight times.

### Discriminating doses

The original bottle assay protocol [9] stresses the use of the assay as a kinetic tool (i.e. response of mosquitoes over time) but this article emphasizes its function as a rapid end-point assay using discriminating doses which kill 100% of susceptible insects in ≤ 1 h. Mortality data between strains of differing susceptibility can then be analysed statistically as a simple comparison of means. The original protocol has confused some local efforts to implement the assay because of the subjective way in which insects are scored alive or dead. The easiest way to avoid any subjectively is simply to score only dead mosquitoes: those that cannot stand, walk, fly or move any of their limbs.

Doses of 0.5, 2, 10 and 25 μg a.i/bottle were tested against *An. darlingi *and *Ae. aegypti *in order to derive a diagnostic dose. These assays were conducted on a laboratory-reared colony of *Ae. aegypti *described above and on *An. darlingi *from the field (Zungarococha) using mosquitoes captured by trained personnel (human landing catch is the standard surveillance tool of the local health authority). The vast majority of Anophelines were captured before they had blood-fed. After the assays, all Anophelines were returned to the laboratory and identified to species. Only *An. darlingi *were collected. Bottles were treated with technical deltamethrin (> 98% a.i) using 95% ethanol (locally sourced) as a solvent. Assays used three bottles for each insecticide concentration and control. Each assay was repeated at least 12 times on four occasions.

### Shelf-life and re-use of pre-prepared bottles

The portability and cost-effectiveness of the bottle assay would increase if pre-treated bottles could be transported to remote sites and re-used a number of times without re-treatment. To evaluate this, bottles were coated with the discriminating dose of 10 μg a.i technical deltamethrin (see above) using 95% ethanol as the solvent (controls consisted of bottles treated with ethanol only). All bottles were stored in the dark under ambient laboratory conditions (27 ± 3°C). At intervals of one, four, seven and 14 days, the lethal effects of these bottles were assessed using *Ae. aegypti*. Another subset was assayed on each of five consecutive days to determine the number of times they could be reused. Fifteen three- to four-day old females, starved of a blood meal for at least 24 hs were exposed in each bottle. Each assay consisted of three treated bottles and three controls and assays were repeated three times.

### Surveillance exercise using the bottle assay

Four rural villages in the Amazon department of Loreto, Peru were selected as study sites (10 - 400 miles from Iquitos by river). All sites are subject to sporadic adulticiding campaigns of Anopheline control and exhibit representative annual malaria parasitaemia indices (APIs) for the region. In 2005, Ullpayacu, Intuto, Libertad and Zungarococha reported APIs of ca. 100, 400, 100 and 50 respectively (Dirección General de Salud Ambiental, unpublished). Pre-treated bottles (10 μg a.i technical grade deltamethrin/bottle using 95% ethanol as a solvent) were transported to each site by local ferry and canoe. Bottles were transported in standard, compartmentalized cardboard boxes and there were no breakages. Mosquitoes were collected between 18:00 - 21:00 by trained local-authority technicians and assays were performed in the shelter of a local health post or school room. Assays concluded before midnight and the team left the site the following morning. All mosquitoes were identified to species. The vast majority were *An. darlingi *or *Anopheles benarrochi*. In some parts of Peru, *An. benarrochi *supplants *An. darlingi *as the main malaria vector [15].

Each test replica utilised 180 females (15 mosquitoes in each of three untreated bottles and nine bottles treated with the diagnostic dose). At all survey sites, assays were repeated twice in the same evening (24 pre-treated bottles, 360 mosquitoes). At one site (Ullpayacu), only 270 mosquitoes were collected and therefore only six bottles at the diagnostic dose were used for each replica. Temperature and relative humidity at the sites varied from 23-25°C and 62-65 RH.

### Comparison of WHO and bottle assays

*Anopheles albimanus *is the only Anopheline to exhibit pyrethroid resistance in Peru [16]. For this reason, that species was chosen for the comparative assays. Blood-fed *An. albimanus *were collected from the fences of sheep and cattle corrals at two sites in the department of Piura. Bottle assays were conducted using bottles pre-prepared with 25 μg a.i of deltamethrin (< 98%) or an equivalent weight of a 5% formulation (K Othrine 50 SC, Bayer). Ethanol (95%) was used as the solvent. 25 μg a.i. deltamethrin/bottle is the dose recommended by CDC to kill 100% of susceptible Anophelines in less than 1 h [14].

The protocol for the WHO assay [17], using impregnated papers treated with 0.05% deltamethrin, requires mosquitoes of known physiological condition, a holding facility suitable for the manipulation of the bioassay equipment and maintenance of the mosquitoes until the 24 h end-point. To accommodate these requirements, mosquito collections for both assays were made the evening before the test and maintained overnight in collecting cups, supplied with sugar solution. Both assays were conducted in parallel on the following morning, at the Centro de Investigación y Capacitación en Entomología, Sullana, Perú.

### Statistical analysis

Data were transformed [arcsin (sqrt p)] for analysis by ANOVA and t-test. Data are presented as back transformed means and 95% confidence limits.

## Results

### Evaluation of locally-sourced solvents and formulations

**Figure 1:**
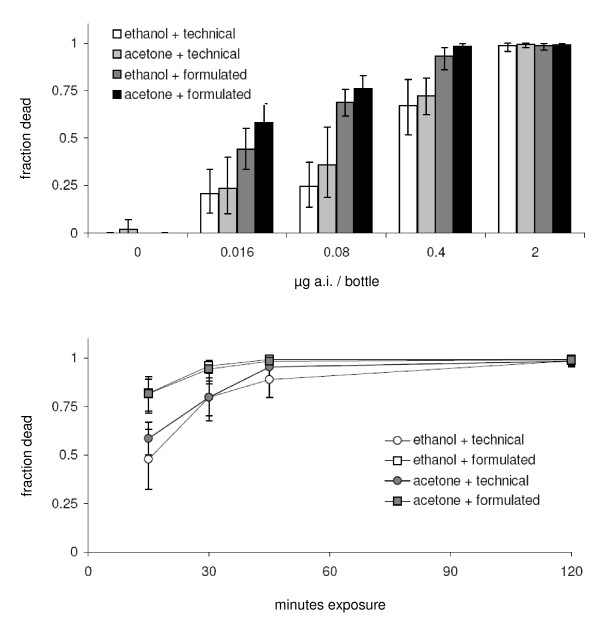
**A. The effect of different solvents and formulations on the mortality of mosquitoes exposed to a range of deltamethrin doses for 2 h [means ± 95% confidence limits]**. **B**. A comparison of time-series relationships for different formulations and solvents at 2 μg/bottle [means ± 95% confidence limits].

Mosquitoes exhibited no differences in mortality associated with the use of different solvents (F > 0.06 < 3.71, p > 0.057 < 0.79). This was true at every time interval (only the 2 h reading is illustrated in Figure 1A). Exposure to formulated material however (a 2.5% wettable powder) gave consistently greater mortality than exposure to technical insecticide (F > 7.4 < 38.9, p < 0.01). This was true at all doses except the highest (2 μg), which after 2 h had killed > 98% of all mosquitoes regardless of solvent or formulation. The time-mortality response was similar between solvents (Figure 1B), but significantly different between technical and formulated compounds.

### Discriminating doses

**Figure 2:**
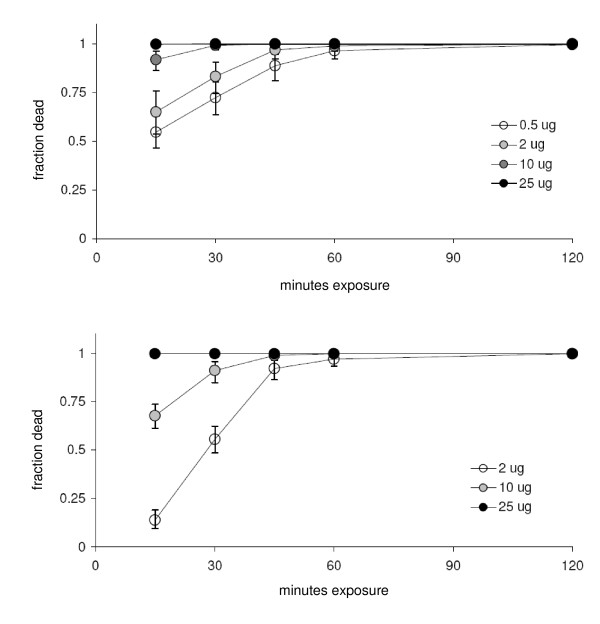
**A. A comparison of *An. darlingi *mortality in response to increasing doses of deltamethrin (μg a.i./bottle) [means ± 95% confidence limits]**. **B**. A comparison of *Ae. aegypti *mortality in response to increasing doses of deltamethrin (μg a.i./bottle) [means ± 95% confidence limits].

For both *An. darlingi *and *Ae. aegypti*, 10 μg a.i deltamethrin per bottle was sufficient to kill 100% of all mosquitoes within 1 h (Figures 2A and 2B). Doses either side of this diagnostic required an endpoint of greater than 1 h, or resulted in such rapid mortality that the presence of moderately resistant individuals might be masked.

### Shelf-life and re-use of pre-prepared bottles

**Figure 3:**
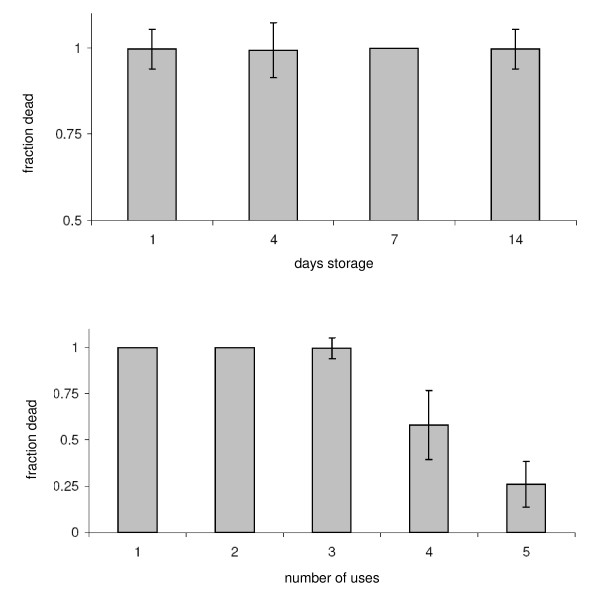
**A. A comparison of *Ae. aegypti *mortality in bottles pre-prepared with the diagnostic dose of (10 μg a.i./bottle) and stored for a number of days [means ± se]**. **B**. A comparison of *Ae. aegypti *mortality in bottles pre-prepared with the diagnostic dose of (10 μg a.i./bottle) and used repeatedly [means ± se].

After 14 days, bottles treated with 10 μg a.i deltamethrin per bottle continued to kill > 99% of all *Ae. aegypti *(Figure 3A). There were no differences between dates (F = 0.36, p = 0.78). Bottles could be used three times before their lethal effects waned (Figure 3B). On the fourth use, mortality fell to 78% overall (F = 21.6, p < 0.001).

### Surveillance exercise using the bottle assay

**Figure 4:**
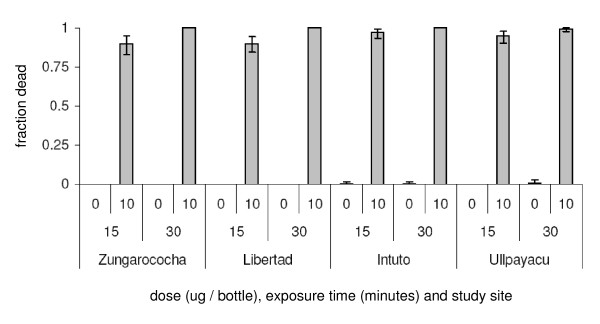
**Susceptibility of *Anopheles *species to the diagnostic dose of 10 μg a.i./bottle**. Mosquitoes were collected at 4 sites in Loreto, and assays were conducted by local technicians using pre-prepared bottles (10 μg a.i/bottle) [means ± 95% confidence limits]. **Footnote to Figure 4 **:Species used in tests - Zungarococha and Libertad: 360 *An. darlingi*; Intuto: 262 *An. benarrochi, 62 An. darlingi, 35 Anopheles oswaldoi, one Anopheles mediopunctatus; *Ullpayacu: *360 An. bennarochi*.

All populations of *An. darlingi *and *An. benarrochi *(both small-bodied Nyssorhynchus mosquitoes) reacted similarly to our diagnostic dose of 10 μg a.i/bottle. After 15 min, average mortality was 85-95%. By 30 min > 99% of all mosquitoes had died (Figure 4). There were no reported problems with the use or efficacy of the pre-prepared bottles. All assays were completed in a single night, using local school rooms or health posts.

### Comparison of WHO and bottle assays

**Figure 5:**
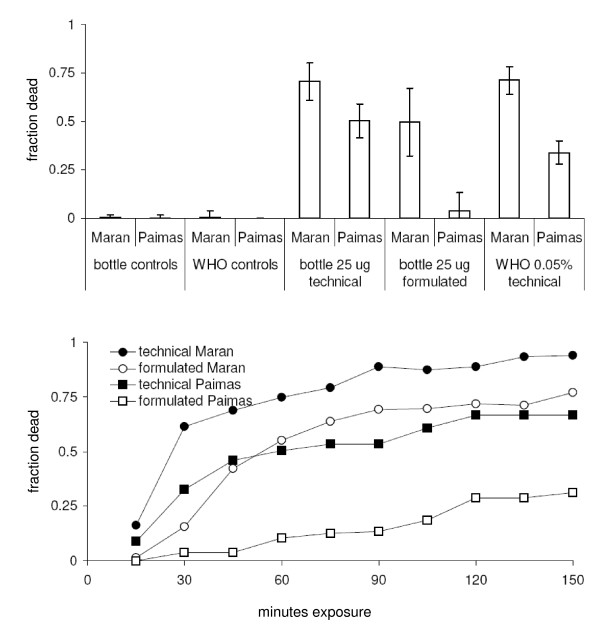
**A. Mortality of two populations of *An. albimanus *in response to the bottle assay (technical and formulated deltamethrin at 25 μg a.i/bottle, end point of 1 h) and the WHO assay (0.05% deltamethrin, end point of 24 h) [means ± 95% confidence limits]**. **B**. Time - mortality curves for *An. albimanus *from Piura in response to the diagnostic dose of 25 μg a.i./bottle (untransformed means).

The WHO assay, using filter papers impregnated with 0.05% deltamethrin (the concentration that kills 100% of susceptible Anophelines in 24 h; [17]) easily distinguished the two populations (Figure 5A). Both were resistant by WHO criteria, but the Maran population was more susceptible than the Paimas population (71 and 34% mortality respectively after 24 h; F = 44.7, p < 0.001).

Figure 5B shows the time-mortality response of the bottle assay over a 2.5 h period. After 45 min, the numbers of mosquitoes dying in both populations began to plateau. This indicated a large proportion of resistant insects. There were large differences in response between the technical and formulated materials (Figure 5A and 5B); for both populations, mortality was lower in bottles prepared with K Othrine 50 SC than in those treated with technical chemical (F = 4.86 p < 0.05, F = 22.8 p < 0.0001 respectively). The use of either technical or formulated material easily discriminated between the populations. After 1 h (Figure 5A) bottles treated with technical deltamethrin caused 71% and 50% mortality for the Maran and Paimas populations respectively (F = 9.81 p < 0.006). For formulated material this was 50% and 4% (F = 16.1 p < 0.001). This 21 to 46% difference in response between the two localities is similar to the discrimination given by the WHO assay (a 37% difference between populations).

## Discussion

The WHO assay is used on a global scale, including the Amazon basin [10,12,16,18-20] and is a familiar and trusted tool. Its components are purchased from a single-source. This requisite exists to remove local error from the assay process, but it can compromise the flexibility and accessibility of the assay. The existing kit for the creation of a baseline characterization of susceptibility allows the assessment of five insecticide concentrations and a control and costs 60 USD, plus 18 USD for each set of impregnated papers [21]. These sums can obviously spiral rapidly with expansions in the surveillance programme. In resource-poor environments, such as Peru, resistance monitoring campaigns are heavily centralized [22,23], a consequence of the value and scarcity of WHO bioassay equipment, the need to plan far in advance in order to ensure a supply of valid insecticide-treated papers, and the lack of suitably trained technicians within local health authorities.

In comparison, the bottle assay has rarely been used to monitor resistance in South American countries [24]. It is in common use elsewhere as a research aid [25-29], but local health authorities around the world need comparative data on the ease and practicability of its implementation. The bottle assay requires generic 250 ml clear glass bottles, disposable plastic pipettes and a quantity of solvent. The original CDC protocol [9] uses acetone to coat the bottles and technical insecticide as the source of active ingredient. Technical insecticide can be expensive to source (e.g. 1 g deltamethrin costs ca. 100 USD) although gram quantities are sufficient to prepare thousands of bottles. In the Amazon, acetone is expensive and its purchase restricted because of its role in the purification of cocaine.

This paper shows that ethanol (95% purity) can be used as an alternative to acetone, and that at least some formulations can be used in place of technical grade insecticides. It was also determined that once treated, pre-prepared bottles that had been capped and stored in the dark at ambient temperatures could be stored for at least 14 days and re-used on three occasions (after three uses they appear to lose effect, presumably due to the redistribution of insecticide caused by contact with mosquitoes, aspirators and moisture from the air or from mouth aspiration). Wherever bottles are to be pre-prepared and stored, similar tests should be used to define their effective shelf lifes.

Ethanol and formulated insecticides are readily available in local public health laboratories. The purchase of six clear 250 ml bottles (available in Peru from a number of suppliers), which will duplicate the capacity of the basic WHO kit outlined above, will cost less than 10 USD. The current study showed that the bottle assay characterized differences in deltamethrin susceptibility between Peruvian *An. albimanus *populations as effectively as the WHO assay. This suggests that it may be a valuable complementary or alternative tool under some conditions. It is important to note that, in our comparisons, a wettable powder formulation was associated with greater mortality than technical deltamethrin, and a suspension concentrate with less. These differences are unsurprising given that the uptake of insecticide is strongly affected by formulation, but it is clear that separate baselines will have to be defined for each one.

The identification of 10 μg a.i. technical deltamethrin as a diagnostic dose for both *Ae. aegypti *and *An. darlingi *in the bottle assay reflects the absence of pyrethroid resistance in either species in the Peruvian Amazon. This is confirmed by the results of the local public health laboratory, who undertook WHO assays in the same year that the bottle assays described in this paper were conducted. They used the same strain of Aedes, and populations of Anophelines from the villages of Libertad, Intuto and Zungarococha. They tested > 400 individuals from each locality and found no survivors at the WHO diagnostic dose [30]. In the surveillance exercise using the bottle assay, a dose of 10 μg a.i./bottle was used to monitor susceptibility in *Anophelines *at these same sites and also in the village of Ullpayacu. This surveillance exercise confirmed the deltamethrin-susceptible status of all Peruvian *An. darlingi *populations. All bottle assays were conducted and completed on field-caught Anophelines, on the night of capture. In comparison, the usual procedure for the WHO assay is to collect the mosquitoes at night, maintain them at a health post or school house until the following day, and then expose them to WHO insecticide-treated papers for 1 hour. Mosquitoes are then transferred to clean holding tubes and held for a further 24 hours until the designated WHO assay endpoint [17]).

The total cost of the bottle assay surveillance exercise, coordinated by the technical team at the public health laboratory in Iquitos, and including all transport, materials and personnel costs, was 1,328 USD. Comparative costs for an identical exercise using the WHO kit are not available but might be considerably greater given the greater cost of the monitoring kits, the increased time needed to conduct the assays, and the centralised nature of the surveillance programme. The costs of a resistance monitoring programme utilising either method will, however, be negligible when contrasted with the economic impacts of vector borne disease and of mosquito control programmes overall. In Peru, 60% of the 87,500 malaria cases reported in 2005 occurred in the Amazon department of Loreto [22]. Each case, including treatment and lost income, has been estimated to cost 250 USD [31]. The total annual investment in vector control (insecticides and operational costs) in Peru is approximately 3.8 million USD (DIGESA, unpublished).

## Conclusion

The bottle assay is a simple, flexible and robust resistance monitoring tool that, at least under the conditions examined here, was able to discriminate between pyrethroid-susceptible and resistant mosquito populations as effectively as the WHO assay. It demands further operational studies regarding its suitability for other species, and other insecticide classes and formulations. One of its main advantages is that it is not dependent on centrally-sourced materials, but this also requires that local laboratories prepare their own insecticide dilutions, treat their own bottles and establish new diagnostic doses for each new formulation that they test. Thus, an element of quality control is lost. Nonetheless, the calculations required and the coating techniques are simple [14] and any local laboratory should be as capable of preparing bottles and identifying the diagnostic doses as the public health laboratory in Iquitos. Pre-prepared bottles can then be transported to other sites under ambient conditions if surveillance is to be carried out at finer local scales.

Given the familiar and established nature of the WHO assay for many health authorities, the bottle assay might initially be best used as a cheap and flexible tool for local resistance surveillance. National coordinating networks might retain the WHO assay as a quality control tool against which to ensure the reliability of the bottle assay results.

## Competing interests

The authors declare that they have no competing interests.

## Authors' contributions

EZ coordinated the assays and managed the logistics of the surveillance programme. RB and EZ facilitated the WHO assays in Piura. MP and WB contributed to the study design and concept. GD oversaw the study and wrote the paper. All authors read and approved the final manuscript.
